# Importance of research for the specialty of Emergency Medicine in India

**DOI:** 10.1186/1865-1380-7-S1-I1

**Published:** 2014-07-25

**Authors:** Kumar Alagappan, Anthony Brown, Latha Ganti, Michelle Biros, Swaminatha Mahadevan

**Affiliations:** 1 Baylor College of Medicine, Houston, TX, USA; 2 University of Queensland, Brisbane, Australia; 3 University of Central Florida College of Medicine, Gainesville, FL, USA; 4 University of Minnesota, Minneapolis, MN, USA; 5 Stanford University, Palo Alto, CA, USA

## 

Emergency Medicine (EM) is a relatively new specialty that is expanding at a phenomenal pace across the world. Within the international community, leaders in medicine, health economics, public health, and government have recognized the importance of developing systems that respond to acute medical and surgical emergencies, and that emergency medicine is a unique discipline that possesses the body of knowledge necessary to respond to these life-threatening crises.

Many countries have already recognized the specialty of EM and offer specific training programs to develop a cadre of physicians with the knowledge and skills to care for patients presenting with emergent medical problems. As India enters the 21^st^ century, Emergency Medicine has now been recognized as its own specialty. This recognition comes with the responsibility for developing a skilled approach to the emergency patient in the Indian setting. The unique issues that surround the Indian patient, from access to care, to cultural issues, to finances, can best be addressed by physicians who work in this environment.

The International Summit on Emergency Management and Trauma (ISEMT 2014) is one of many new Emergency Medicine conferences that has encouraged India’s core of EM physicians and researchers to address novel clinical issues, collect data, and share these findings amongst themselves. By conducting research in India in the field of emergency medicine, India will be better poised to address the needs of the general population in the coming years.

The abstracts selected for presentation at the ISEMT2014 give an invaluable insight into the breadth and diversity of contemporary emergency and trauma care in India. The reason they are so important is that all research is about testing and showing that each of us is practicing the science of medicine to the best of our abilities. Whilst the case report is a popular first step, it cannot demonstrate cause and effect. Retrospective analysis begins to align information with outcome, but is still fraught with many biases. Prospective data collection is the most worthy goal, aimed at testing a hypothesis with exact methodology around sample size, variables, confounders and statistical analysis. These abstracts show clear and purposeful steps in the right direction.

Only half of the submitted ISEMT 2014 abstracts were chosen for presentation, and they were selected by a panel of distinguished judges who serve on the editorial board of several prominent international EM journals. It is our sincere hope that these abstracts will be published in peer-reviewed journals around the world and begin to define emergency medicine research in India. We encourage the great work that is now being done in the field of EM, and look forward to even greater collaboration and research in the near future.

